# GradWise: A Novel Application of a Rank-Based Weighted Hybrid Filter and Embedded Feature Selection Method for Glioma Grading with Clinical and Molecular Characteristics

**DOI:** 10.3390/cancers15184628

**Published:** 2023-09-19

**Authors:** Erdal Tasci, Sarisha Jagasia, Ying Zhuge, Kevin Camphausen, Andra Valentina Krauze

**Affiliations:** Radiation Oncology Branch, Center for Cancer Research, National Cancer Institute, National Institutes of Health, Building 10, Bethesda, MD 20892, USA

**Keywords:** brain tumor, glioma, grading, feature selection, pattern recognition, classification, data mining, molecular data, oncology

## Abstract

**Simple Summary:**

Glioma tumor aggressiveness is expressed as tumor grading which is crucial in guiding treatment decisions and clinical trial participation. Accurate and standardized grading systems are essential to optimize care and improve outcomes. However, integrating molecular and clinical information in the grading process has the potential to expose molecular markers that have gained importance in understanding tumor biology as a means of identifying druggable targets. In this study, a novel approach called GradWise is introduced with the goal of enhancing feature selection performance while employing various machine learning models of glioma grading. GradWise combines a rank-based weighted hybrid filter (mRMR) and an embedded feature selection method (LASSO) to select the most relevant features from clinical and molecular predictors and was evaluated using two commonly employed public biomedical datasets, TCGA and CGGA, utilizing two feature selection methods and five supervised models. The findings support existing evidence and provide pioneering results for glioma-specific biomarkers, highlighting the effectiveness of the approach and future directions for biological mechanisms of glioma progression to higher grades.

**Abstract:**

Glioma grading plays a pivotal role in guiding treatment decisions, predicting patient outcomes, facilitating clinical trial participation and research, and tailoring treatment strategies. Current glioma grading in the clinic is based on tissue acquired at the time of resection, with tumor aggressiveness assessed from tumor morphology and molecular features. The increased emphasis on molecular characteristics as a guide for management and prognosis estimation underscores is driven by the need for accurate and standardized grading systems that integrate molecular and clinical information in the grading process and carry the expectation of the exposure of molecular markers that go beyond prognosis to increase understanding of tumor biology as a means of identifying druggable targets. In this study, we introduce a novel application (GradWise) that combines rank-based weighted hybrid filter (i.e., mRMR) and embedded (i.e., LASSO) feature selection methods to enhance the performance of feature selection and machine learning models for glioma grading using both clinical and molecular predictors. We utilized publicly available TCGA from the UCI ML Repository and CGGA datasets to identify the most effective scheme that allows for the selection of the minimum number of features with their names. Two popular feature selection methods with a rank-based weighting procedure were employed to conduct comprehensive experiments with the five supervised models. The computational results demonstrate that our proposed method achieves an accuracy rate of 87.007% with 13 features and an accuracy rate of 80.412% with five features on the TCGA and CGGA datasets, respectively. We also obtained four shared biomarkers for the glioma grading that emerged in both datasets and can be employed with transferable value to other datasets and data-based outcome analyses. These findings are a significant step toward highlighting the effectiveness of our approach by offering pioneering results with novel markers with prospects for understanding and targeting the biologic mechanisms of glioma progression to improve patient outcomes.

## 1. Introduction

Tumor grading is the classification of tumor aggressiveness determined via the evaluation of tumor characteristics with additive molecular features under the microscope [1]. Gauging the aggressiveness of a tumor, in this case, glioma represents a surrogate for anticipatory biological behavior. A sense of how the tumor will behave with or without treatment is crucial for decision-making at diagnosis, which connects to managing, monitoring, and treatment planning [2]. Gliomas are the most common primary brain tumor originating from glial cells and can be highly aggressive, progressive, and neurologically devastating [3,4]. Currently, according to the World Health Organization (WHO) guidelines, gliomas are categorized into low-grade (LGGs) and high-grade gliomas (HGGs), with glioblastoma multiforme (GBM) being the most aggressive and invasive. Treatment options and survival rates are highly dependent on tumor grade.

The current approach for treating gliomas is primarily determined by the grade of the tumor and typically involves maximal surgical removal followed by radiation therapy (RT) [2,4,5]. Additionally, patients may receive systemic treatment in the form of chemotherapy using temozolomide (TMZ) administered concurrently or sequentially with either sequential PCV or PC (Procarbazine, CCNU with or without vincristine) as an alternative [2,4,5]. Typically, the diagnosis of glioma is made by obtaining tissue for pathological examination with molecular alterations being increasingly important for CNS tumor classification [6,7,8]. The isocitrate dehydrogenase (IDH) mutation is now more routinely employed as a molecular marker, given its prognostic value [4,9,10], but is limited by the associated costs and turnaround time of molecular testing, with p.R132H-specific IDH1 immunohistochemistry costing USD 135, single-gene sequencing costing USD 420, and next-generation sequencing costing USD 1800 [9] and the time required for analysis ranging from approximately two days for immunohistochemistry to up to 14 days for next-generation sequencing [9]. IDH mutation vs. IDH wild-type confers superior prognosis, particularly when accompanied by 1p19q co-deletion altering the management of non-GBM gliomas in terms of type and timing of systemic management, while GBMs are treated with standard-of-care concurrent chemo-irradiation irrespective of IDH status. However, despite the superior prognosis conferred by IDH mutation, there is an ongoing lack of clarity as to the mechanism by which IDH mutation connects to the prognosis conferred to patients. There is an ongoing need to identify markers that allow for glioma grading via linkage to biological mechanisms that can be exploited to alter outcomes by modulating tumor resistance and response.

The tumor grading process incorporates clinical features such as age and gender [11], but publicly available datasets lack robust higher-level clinical annotation, which limits the connection between relevant molecular features and clinical data. This gap could be bridged by increasing reimbursement for molecular testing, which could promote more widespread use and benefit value-added care [4]. Therefore, selecting the best molecular and clinical markers that distinguish between tumor grades would not only reduce costs to healthcare systems and patients but also enhance tumor grading performance. This improvement in performance would enable the selection of significant molecular features for future research and testing of novel targeted agents [4]. However, given the partial nature of available molecular information, optimal utilization of such data would require computational analysis. Hence, feature selection plays a vital role in this context.

The feature selection stage is a critical step in machine learning, where the objective is to select a subset of relevant features among all features that improve the accuracy of the model while reducing complexity. Feature selection is generally utilized for data analysis, pattern recognition, data mining, and machine learning tasks. This process aims to improve performance (e.g., tumor grading) and classification accuracy rate and provide computational efficiency by removing irrelevant or redundant features and reducing the dimensionality of data [12,13,14,15,16,17]. There are various feature selection methods available, such as filter methods, wrapper methods, and embedded methods [12,18], each with its own advantages and limitations. The selected features can be used for further analysis, such as identifying biomarkers, developing predictive models, and gaining insights into the underlying biology of the disease.

Generally, feature selection methods are applied to training-test sets to determine the relevant feature subset, or these methods are aimed at reducing costs and improving classification evaluation results. If there is no training-test set separation, we should use these methods with the cross-validation technique for validation purposes. However, identifying molecular feature names is crucial to being able to leverage the identified features biologically and clinically. To this end, feature-weighting, counting, or rank-based approaches can be used [19]. While several markers are being used in the clinic and additional markers are being proposed, given evolving research, there is currently no robust biomarker list or panel that defines glioma grading and employs data from TCGA and CGGA, which are the most commonly available and utilized datasets of clinical and molecular data. In this study, the goal was to identify the most important, discriminative, and likely optimal molecular and clinical features for glioma grading by using a novel application of a hybrid rank-based filter and embedded feature selection based-method (GradWise) and five supervised learning models taught with TCGA and CGGA glioma data and to link the results to described mutations in glioma and novel biological applications.

The main contributions of our study are summarized as follows: 

Our study proposes the first application and method that employs a rank-based hybrid feature selection method for feature selection and supervised machine learning models to improve glioma grading.

- We combine the advantages of various feature selection methods via a rank-based feature-weighting approach for glioma grading on two commonly used glioma datasets (TCGA and CGGA).
- We utilize feature-weighting to determine which features are significant, enabling validation of this method for glioma grading tasks.
- We conduct a comprehensive computational analysis comparing our feature selection methods, given that these are two commonly employed glioma datasets that share similarities but also exhibit differences.
- Our objective is to determine the optimal combination of feature subsets and learning models during the feature selection stage, aiming to achieve high accuracy with a minimal number of features while accounting for dataset variability in large-scale datasets. This approach seeks to provide accurate results that can be transferred and applied effectively across different scenarios.
- We introduce a TCGA- and CGGA-specific shared feature set and connect identified features for glioma grading with described mutations in glioma and identify potential mechanistic implications for progression to higher grade.

The remaining sections of our study are structured as follows: In Section 2, we provide an overview of the employed methodology and explain the related feature selection, feature-weighting methods, and classification models for glioma grading. In Section 3, we describe the experimental procedures, datasets employed, and evaluation metrics and provide comprehensive experimental results with discussions. Finally, Section 4 encompasses the study’s conclusion, a discussion of the results, and potential avenues for future research.

## 2. Methods

In this section, we present a concise summary of the feature selection and weighting architecture that is being proposed for glioma grading via clinical and molecular characteristics. The subsequent subsections outline the methods used for feature selection, feature-weighting, and classification in this study.

### 2.1. The Utilized Methodology for Glioma Grading

**Figure 1:**
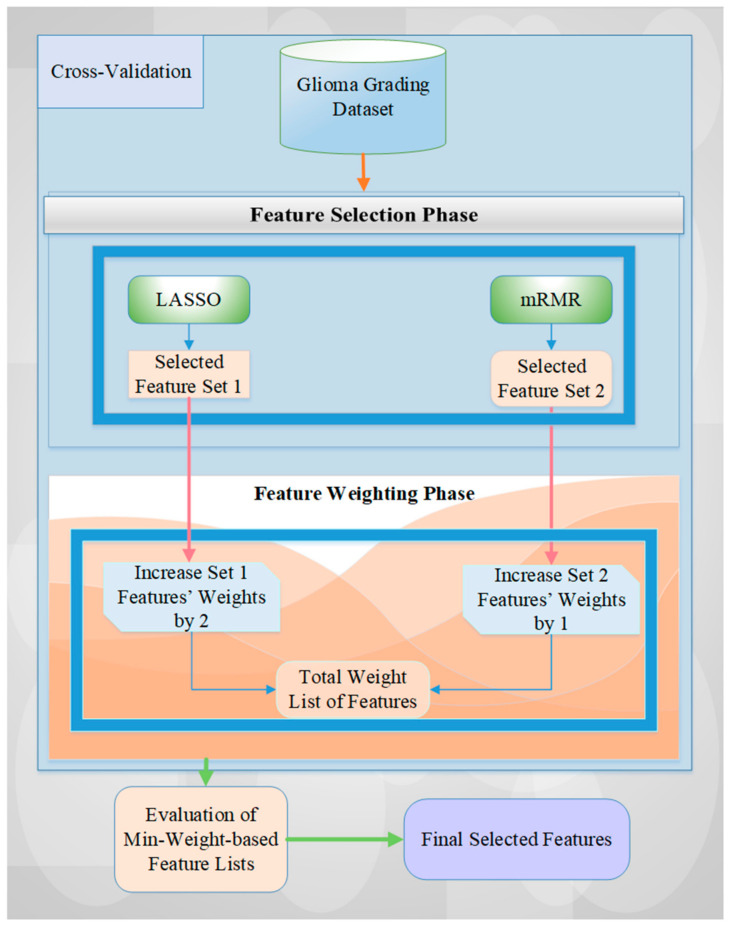
A detailed overview of the proposed methodology.

In this study, we employ a hybrid method for weighting and selecting of features based on ranks [19] which can be used to categorize glioma grades. Our used methodology consists of two main phases: (i) feature selection (FS) and (ii) feature-weighting (FW) [19]. Figure 1 and Table 1 provide a sample algorithmic diagram of our utilized architecture and related processes, showcasing the two feature selection methods used: LASSO and mRMR. 

At the outset, all clinical and molecular features are fed into the feature selection (FS) model using a cross-validation technique. For each fold, the feature sets selected by the two FS methods are saved, and their counts are increased based on the corresponding weights assigned by the rank-based approach. Next, the minimum weight-based feature list is evaluated with all weight values. In the final stage, we obtain the final selected feature list by evaluating all weight values and identifying those with the highest accuracy rate.

In other words, LASSO and mRMR feature selection methods are employed to select features for each fold of the cross-validation of the dataset based on clinical and molecular predictors. After selecting the features, their weights are increased according to their rank-based importance level, which is determined based on their performance results in terms of accuracy (see Table 2 and Table 3). Specifically, the weight of a feature selected by LASSO is increased by two, while that of a feature selected by mRMR is increased by one. If the same feature is chosen by both methods for all five folds of cross-validation, its weight is 15, which is the maximum weight value for 5-fold cross-validation. On the other hand, if a feature is not selected by either FS method for a given iteration, we assign it a weight of 0. However, we ensure that the minimum weight value of a selected feature is identified as at least 1 to use all selected features of all cross-validation iterations for the experimental results.

Following the feature selection and feature-weighting stages, we obtain total weights with the corresponding feature lists and evaluate the minimum weight-based feature lists to select the final features for all weight values. To illustrate this process, consider an example. Suppose we determine the minimum weight as 12 for our study. In this case, we can select the features with weight values of 12, 13, 14, and 15 as the final feature set. We evaluate these weight values based on their performance results in terms of accuracy rate and identify the minimum weight value that achieves the highest accuracy rate with the minimum selected number of features for all values in the dataset.

### 2.2. Feature Selection and Feature-Weighting

The aim of feature selection methods is to reduce the dimensionality of data space by obtaining a suitable feature subset from all features. This process eliminates redundant, insignificant, or irrelevant features and yields better model interpretation and diagnosis capability, thus accelerating prediction speed and reducing the time requirement of the training stage of the machine learning model [12,20,21]. Additionally, feature selection methods deal with high-dimensional data, computational and storage complexity, data visualization, and high-performance issues for machine learning-related problems in real-world applications [12,22]. Feature selection methods are generally classified into three categories, depending on the evaluation metric of the feature subset: filter, wrapper, or embedded methods [18]. Univariate filter and multivariate filter FS methods are two subcategories of filter methods that consider relationships between features and/or between features and the target/class or output variable [19]. In this study, we utilized a multivariate filter FS method called minimum redundancy maximum relevance (mRMR) and an embedded-based FS method called LASSO to select the clinical and molecular features of glioma patients’ data. We chose to avoid the computational load of the wrapper FS method and the dependence on model-specific features associated with this approach in order to enhance the transferability of our used approach. In the feature-weighting stage, the importance level of each selected feature in discriminating pattern classes is typically represented by a weight value, which can be added or multiplied to feature values [19,23]. In this study, a rank-based feature-weighting approach was adopted. The two-feature selection (FS) methods, LASSO and mRMR, were ranked based on their performance results in terms of the accuracy rate as described in [19]. 

### 2.3. Classification

The classification phase is a fundamental task in machine learning that involves assigning predefined labels or categories to input data points based on their features. The goal of classification is to build a predictive model that can accurately classify new instances into their appropriate classes. Classification algorithms learn patterns and relationships from labeled training data, enabling them to make predictions from unlabeled data. Commonly used classification algorithms include k nearest neighbors, logistic regression, support vector machines, random forests, and AdaBoost. We briefly describe these learning models in the subsequent subsections in our previous work [4].

## 3. Experimental Work

This section describes the experimental processes and environment, relevant parameters, and performance metrics and explains our clinical and molecular dataset for glioma grading. Subsequently, we provide a detailed presentation of our comprehensive computational results, highlighting the impacts of various feature selection methods.

### 3.1. Experimental Process

To implement the proposed methods in this study, we employed the experimental process previously described [4,19]. 

We used a preprocessed glioma grading dataset with clinical and molecular features and performed a z-score normalization approach to age feature values before the feature selection and classification phases. We also employed a 5-fold cross-validation technique during the feature selection and weighting processes to ensure robustness. This approach allowed us to evaluate the performance of the utilized learning models and obtain average performance results, enhancing the reliability of our findings. For the evaluation of the learning models in this study, the GBM class was designated the positive class, while the LGG class served as the negative class.

Both the mRMR and LASSO methods were employed at the hybrid feature selection stage. In the mRMR-based feature selection, a heuristic value was utilized by taking the logarithmic value of the total number of features (i.e., ⌈log2(Total Number of Features)⌉ = round of log2(Total Number of Features(23)) = 5) to determine the number of selected features. For LASSO-based feature selection, a 10-fold cross-validation was performed to determine the optimal alpha parameter value across iterations and identify the number of selected features.

In the feature-weighting stage, a rank-based approach was utilized, where the weights of features were determined based on the performance results of the feature selection methods, specifically the accuracy rate. To identify the final selected feature set, various minimum weight values ranging from 15 to 1 for 5-fold cross-validation were tested to find the subset of features that achieved the highest accuracy rate while using the smallest number of features.

### 3.2. Dataset

We employed the Cancer Genome Atlas (TCGA) [24] and the Chinese Glioma Genome Atlas (CGGA) [25] databases, which are widely used for analyzing brain tumors (specifically glioma), to assess our employed methodology for rank-based feature-weighting and selection processes. The original TCGA dataset is described in our previous work [4] and Table 2 with the preprocessed TCGA dataset for glioma grading available on the UCI Machine Learning Repository [24]. The CGGA dataset consists of 22 features (one fewer than TCGA) with the same characteristics described in Table 2. The dataset query and storage operations were facilitated via the NIDAP environment [26]. The quantitative description of gene expression (mutated/not mutated frequencies) in TCGA is presented in Supplementary Figure S1 and was described for TCGA by Yan et al. [27]. Quantitative description is available for CGGA at http://www.cgga.org.cn/analyse/WEseq-data-oncoprint-result.jsp, and was described by Hu et al. [28]. 

### 3.3. Performance Metrics

To assess the performance of the utilized methodology in feature selection and classification, six evaluation metrics were used: classification accuracy (ACC), area under the ROC curve (AUC), F-measure (F1), precision (PRE), recall (REC), and specificity (SPEC) [29]. These were described in detail in our previous work [4]. 

### 3.4. Computational Results

In this subsection, we present the experimental results showcasing the impact of the feature selection and feature-weighting approaches on the performance analysis of the models investigated in this study. The most optimal results are indicated by bold values. # represents the number. The best result for each method is highlighted in bold.

#### 3.4.1. The Effects of Using Feature Selection Methods

During the initial phase, we conducted experiments using five supervised learning models to examine the potential benefits of feature selection (FS) techniques on the glioma grading datasets. Table 3 and Table 4 present the average performance results of these models (i.e., 5-fold cross-validation) on TCGA and CGGA datasets in terms of accuracy rate (%).

The findings in Table 3 and Table 4 demonstrate that the supervised learning models with the LASSO method generally achieved better results compared to the mRMR and no feature selection methods. Using the LASSO FS method, we obtained the best accuracy rate value of 87.007 with the SVM model, while without FS, the best accuracy rate value of 86.769 was achieved with the same learning model, according to Table 3 results. The LASSO FS method also provided better results from the CGGA dataset compared to the no FS method in terms of accuracy rate (see Table 3). This is depicted in dark green in Table 4. The LASSO method has three higher performance results than the best accuracy rate value (i.e., 76.570%) of no FS method result on the CGGA dataset. Additionally, the mRMR method yielded accuracy rate values of 85.935 and 76.933 for the TCGA and CGGA datasets, respectively. Following this stage, in which we obtained these results, we proceeded to the next level of the FS process, which involved assigning corresponding ranks to these FS methods, a process known as feature-weighting. We selected the LASSO FS method as the more significant method with respect to the results obtained from Table 3 and Table 4 to assign ranks to the corresponding methods (increasing feature weight value by two and one for the LASSO and mRMR FS methods, respectively).

#### 3.4.2. The Effects of Using LASSO and mRMR Feature Selection and Feature-Weighting Methods

**Figure 2:**
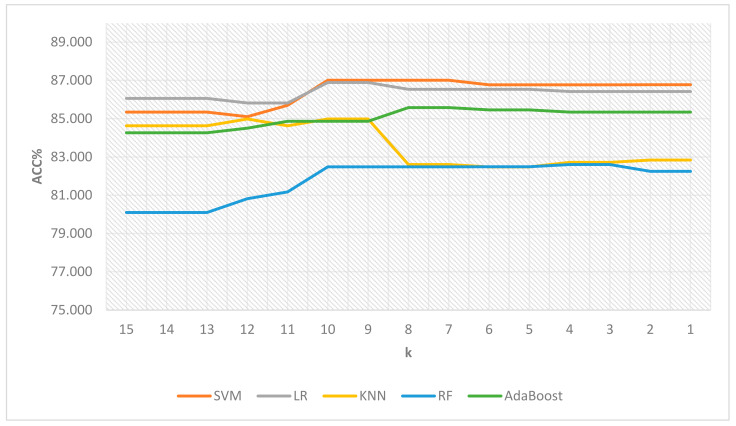
Comparative illustration of feature-weighting and selection results on the TCGA dataset.

**Figure 3:**
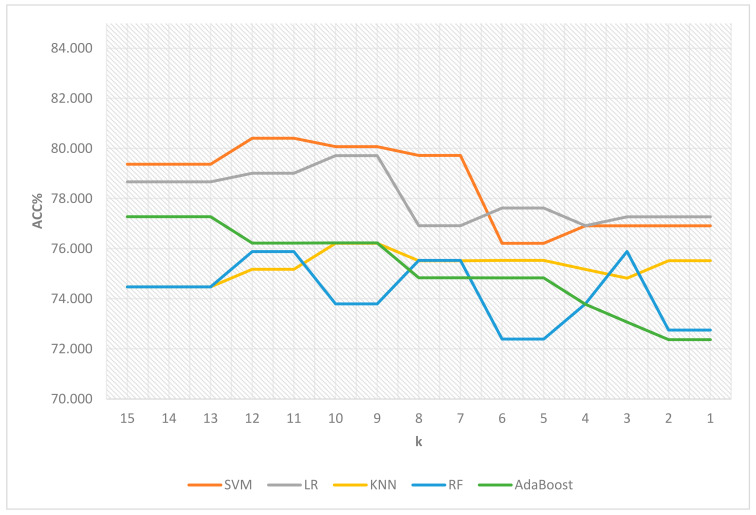
Comparative illustration of feature-weighting and selection results on the CGGA dataset.

After the initial evaluation of features and assigned ranks based on their performance results, the related computational results obtained via the utilization of both LASSO and mRMR-based feature selection (FS) with weighting methods are presented in Table 5 and Table 6 and Figure 2 and Figure 3, which denote the mean accuracy rate values obtained using 5-fold cross-validation. k represents the minimum weight value. 

As seen in the results given in Table 5, the most optimal outcome was characterized by an accuracy rate of 87.007, a minimum weight value of 10, and the selection of 13 features using the support vector machine model. The selected feature names for the best result from the TCGA dataset are as follows: ‘**CIC**’, ‘**Age**’, ‘**IDH1**’, ‘**PTEN**’, ‘**ATRX**’, ‘**PIK3R1**’, ‘**NF1**’, ‘**IDH2**’, ‘**GRIN2A**’, ‘**NOTCH1**’, ‘**TP53**’, ‘**EGFR**’, ‘**MUC16**’. It is noteworthy that the number of selected features remained constant for both minimum weight values of 10 and 9. Consequently, the selection of the minimum weight value did not affect the results. However, in the scenario where the accuracy rate remains unchanged while different numbers of features for various weight values result, the maximum value could have been assigned as the minimum weight value to facilitate the selection of the minimum number of features. The second-best different result achieved the same accuracy rate of 87.007 with a minimum weight value of 8 and the selection of 18 features using the support vector machine. Figure 2 also depicts the line chart for the comparative illustration of feature-weighting and selection results on the TCGA dataset.

As can be seen from Table 6, the best performance results had an accuracy rate value of 80.412 in conjunction with the minimum weight value of 12 and the number of selected features as 5, employing the support vector machine classifier on the CGGA dataset for the glioma grading task. The second-best different result achieved an accuracy rate value of 80.073, with a minimum weight value of 10 and the selection of 8 features, using the support vector machine. The following feature names were selected for the optimal result obtained from the CGGA dataset: ‘**IDH1**’, ‘**Age**’, ‘**PTEN**’, ‘**PDGFRA**’, and ‘**NF1**’. Additionally, Figure 3 is a line chart that visually represents and compares the results of feature-weighting and selection from the CGGA dataset.

By employing this approach, it was possible to identify and select shared clinical and molecular predictors from the initial set of 22 or 23 features depending on the dataset used (i.e., TCGA or CGGA) in the glioma grading dataset. These selected and shared four features have the following names: ‘**IDH1**’, ‘**Age**’, ‘**PTEN**’, and ‘**NF1**’.

#### 3.4.3. Other Performance Results Based on Feature Selection and Weighting Process

We have obtained comprehensive computational results by employing a hybrid feature selection and weighting, taking into consideration six performance metrics: accuracy rate (ACC) %, area under the ROC curve (AUC), F-measure, precision, recall, and specificity. Table 6 and Table 7 present the detailed average performance results on the TCGA and CGGA glioma grading datasets with/without feature selection and weighting. The green indicator shows that feature selection and weighting give better results than not applying the feature selection method, while the red indicator represents worse results in the same situation. 

As shown in Table 7 and Table 8, since we focused on obtaining a high accuracy rate from the best-supervised learning model by using feature selection and weighting approaches in this study, support vector machine with FS and FW provides higher accuracy rate values than no FS method. Regarding Table 7 and Table 8, the green color in cells means that the result after FW and FS is higher than the result without FS. Otherwise, the color is orange in cells. The SVM model achieved the highest values on the TCGA dataset, yielding 87.007%, 0.855, and 0.915 for ACC, F-measure, and recall, respectively. The LR model had the highest values for AUC and precision, namely 0.923 and 0.808, respectively, and KNN also yielded the highest value for specificity on the TCGA dataset, namely 0.846. Ash shown by the CGGA dataset-based results (see Table 7), the SVM model on the CGGA dataset achieved the highest values, yielding 80.412%, 0.815, 0.679, 0.807, and 0.913 in terms of ACC, AUC, F-measure, precision, and specificity, respectively. The RF model had the highest value, namely 0.610, in terms of recall on this dataset for this study as well.

#### 3.4.4. Comparison with the Related Methods for Glioma Grading

In this subsection, we compare the performance on the two datasets used of the utilized method of feature selection with another related method from the literature for glioma grading tasks with molecular and clinical characteristics.

In Table 9, the accuracy rates are displayed as percentages along with the number of selected features for each method/dataset combination. Our method surpasses all its competitors by selecting 13 features for the TCGA dataset and 5 features for the CGGA dataset. Taking into account the obtained accuracy rate values, our method also outperforms its competitor [4] with 80.412% from the CGGA dataset. 

Table 9 gives the significant differences between our approach and those of [4] while noting their respective advantages as well. Since we focused on determining feature names with the minimum number of selected features and maximum accuracy rate by using both feature-weighting and selection strategy in this study, our performance results (i.e., ACC) can be lower than the related method’s (e.g., [4]) results (e.g., TCGA dataset). However, our methodology presents more robust, effective, realistic results and identified feature names for the related classification task. The utilization of our method, which incorporates feature-weighting and selection approaches, led to a substantial enhancement in identifying the names of the final selected feature set in our biomarker discovery process.

## 4. Discussion

**Figure 4:**
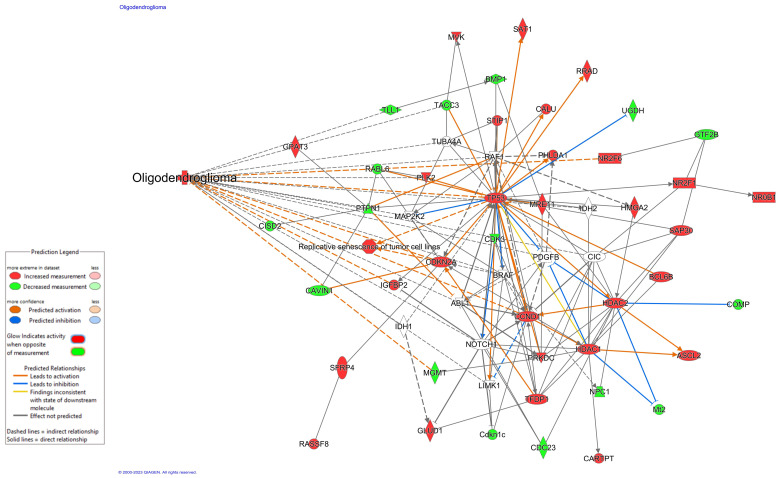
Network output of the 12 GradWise identified molecular features with oligodendroglioma annotation in IPA ((QIAGEN Inc., https://www.qiagenbioinformatics.com/products/ingenuitypathway-analysis) accessed on 6 September 2023) [41]. Several identified features associated with progression to a higher grade and complex biological interplay do not currently exhibit known biological measurement (NOTCH1, PDGFRA, IDH1/2, CIC). GRIN2A and MUC16 did not map in this framework, supporting a more novel role in glioma biology.

Based on the empirical findings from our rank-based weighted hybrid filter and embedded feature selection methodology applied to the TCGA and CGGA datasets with molecular and clinical characteristics, several insights emerged. Our utilized feature selection method demonstrated superior performance in terms of the number of selected features as compared to our previous related method [4] when applied to the same datasets. Since this hybrid method harnesses the advantages of two popular and effective feature selection methods, we hypothesize that it generates superior results as compared to the individual selection methods employed in isolation. This is, however, a novel application to this setting (glioma) and these datasets (TCGA/CGGA), and comparison with the results of other studies is yet limited. In a recent benchmarking study of feature selection strategies in multi-omics data, wherein 15 cancer multi-omics datasets were employed to compare four filter methods, two embedded methods, and two wrapper methods with respect to their performance in relation to the prediction of a binary outcome, the authors found that the feature selection methods mRMR, the permutation importance of random forests, and the Lasso method tended to outperform the other methods [30]. Bhadra et al. compared five widely used supervised feature selection methods (mRMR, INMIFS, DFS, SVM-RFE-CBR, and VWMRmR) for multi-omics datasets from a multi-omics study of acute myeloid leukemia (LAML) from TCGA to successfully identify gene signatures in each data subset [31]. Empirical results suggest that our feature selection and weighting methodology with supervised learning models holds promise for glioma grading tasks. By employing two different feature selection methods and five individual learning models using rank-based weighting strategies, we achieved optimal results in this study. Our methodology also generally outperformed the results of using a no feature selection method when tested on two datasets with different model schemes. A pivotal objective of this study was to determine the names of the final features in the set by assigning ranks and weights to the corresponding methods according to their importance level in terms of accuracy rate compared to the no FS methodology. Although the selections by the best-supervised learning models and features varied across the datasets, we can conclude that our proposed approach [19] yields more robust and effective results compared to our previous feature selection approach [4]. We anticipate that, as integral sources of shared molecular data, TCGA and CGGA will continue to evolve with increasing number of features, and we hypothesize that providing a method that allows for the selection of the most biologically relevant TCGA and CGGA features, the future cost of molecular characterization may be reduced with increasing prediction performance and potential to examine biological pathways of glioma progression at a higher grade. To address the challenge of having a limited number of cases with high-dimensional features, we employed a 5-fold cross-validation technique to mitigate bias. By combining the strengths of both filter and embedded popular feature selection (FS) methods, we achieved highly effective results, even in the presence of high-dimensional features and the complexity of the problem. It should be noted that the choice of the most suitable feature subset may differ based on the feature selection method(s), parameters, heuristics, data type, and dataset size, as there is no universally optimal method that applies to all situations (as exemplified by the ‘no free lunch theorem’) [19]. We acquired thirteen and five significant clinical and molecular biomarkers for TCGA and CGGA datasets, respectively. Differences in identified features between TCGA and CGGA can be attributed to the number of patients, the number of features, data distribution, and characteristics in each set. Four features were shared among TCGA and CGGA: **Age, IDH1, PTEN, and NF1**. This indicates that given this input data, these are the currently most robust if not the most informative markers of glioma grading. The shared features align with existing data surrounding the distinction of diagnostic labels in glioma. Age as the sole shared clinical feature between TCGA and CGGA reflects tumor subtype distribution, which is distinctive between lower-grade gliomas occurring more often in younger patients in contrast to high-grade gliomas, including GBM occurring in older patients. IDH status as a co-localizing feature of more favorable biological behavior separates lower grade from higher grade glioma due to its association with LGG as compared to GBM, of which only approximately 10% are IDH mutated [32,33]. PTEN alteration is more associated with aggressive biological behavior and higher-grade glioma [34,35]. The role of NF1 is an equally well-recognized mutation in glioma, albeit not as common [36]. The shared features thus validate the method based on existing literature evidence (see Table 10). The additional features identified in TCGA (**CIC, ATRX, PIK3R1, IDH2, GRIN2A, NOTCH1, TP53, EGFR, MUC16**) and CGGA (**PDGFRA**) provide interesting avenues for analysis of progression to a higher grade in glioma via linkage to known signaling pathways of tumor progression and treatment resistance (Table 1, Figure 4). In particular, MUC16, also known as CA-125, merits additional investigation given its emergence as a distinctive grading feature, since current literature supports this marker in ovarian cancer with clinical use; however, it is identified as mutated in only a relatively small percentage of gliomas [37]. Recent evidence supports its role in tumor grading and prognosis [38], and it carries mechanistic implications via linkage with PDGFRA, a feature also identified in the current study and interestingly identified in CGGA [39]. Connecting PTEN, ATM, and p53, the feature GRIN2A, only described to date in a small minority of GBMs, merits further study as a marker of possible genetic evolution to a higher grade and post-therapeutic adaptation [35]. Similarly, CIC emerged in this method as a distinctive marker. It has been reported in 20% of LGGs [32]; however, CIC protein instability has been associated with tumorigenesis in GBM [40]. The identification of features such as MUC16 (already in use in the clinic albeit not in the glioma setting) and GRIN2A and CIC, both relatively novel, as evidenced by current ingenuity pathway analysis (IPA) (Figure 4) [41], is not currently employed in the clinic but shows promise in analyzing mechanistic progression to higher grade and showcasing the clinical promise of novel applications such as GradWise as potential tools to identify novel biomarker in existing datasets such as TCGA and CGGA. The reality in the clinic is that multiple loci of different molecular subtypes may be present in tumors, complicating diagnosis (Supplementary Figure S2). The method in this study may advance diagnostic capabilities by leveraging the complex feature composites of several markers and molecular subtypes to match them to the most appropriate diagnostic code. This aspect will be further improved by incorporating progression and survival outcomes as well as complex DNA methylation analysis, which is subject to implementation in multidisciplinary pathology discussions and future directions. The limitations of the study include the small scale of data and low quantity of features. TCGA resulted in several more distinctive grading features compared to CGGA, which indicates dataset-dependent limitations grounded in tumor heterogeneity and class imbalance. 

## 5. Conclusions and Future Work

This study introduces GradWise, a novel application of a rank-based weighted hybrid filter and embedded feature selection method employing LASSO and mRMR-based feature selection and weighting methods for glioma grading. The results demonstrate that the method is effective in identifying features representative of tumor grade and is in agreement with existing evidence, and it thus can serve as a framework for feature selection, classification, and pattern recognition towards value-added care, particularly in the context of molecular, clinical, and proteomic markers, while enhancing the predictive performance of models. The exploration of higher-dimensional biomedical datasets, including proteomic or metabolomic data, suggest future directions of this study to further validate this method. Future directions include the aggregation of additional datasets, including clinical, imaging, and omic data, with higher-dimensional features. This will allow us to further leverage and compare the performance and validation of GradWise against other approaches and explore the use of alternatives or combinations of the ensemble machine learning predictors to improve performance results for specific large-scale medical data scenarios.

## Figures and Tables

**Table 1:** The related textual representation of an employed scheme for the proposed GradWise approach.

1. Input: Clinical and molecular predictors with labels
2. Feature Selection with cross-validation: For each fold: - Choose supervised models (e.g., KNN, random forest, SVM, etc.) - Apply the mRMR (i.e., multivariate filter) FS method - Apply the LASSO FS (i.e., embedded) FS method - Select the most relevant features - Apply feature-weighting (increase the selected features’ weights based on their ranks) 3. Try all weights with all machine learning models to determine the maximum accuracy rate with the minimum number of features 4. Evaluation: - Use TCGA and CGGA datasets - Calculate performance metrics (e.g., accuracy rate) 5. Results: - Obtain accuracy rates and feature counts for TCGA and CGGA datasets - Identify shared biomarkers for glioma grading 6. Conclusion: - Highlight and discuss the potential advantages of GradWise approach - Provide pioneering results for glioma-specific biomarker research and conceptualize findings given existing evidence for driver mutations and progression to a higher grade in glioma.

**Table 2:** The related features and class information for the datasets employed. TCGA has 23 features (3 clinical, 20 molecular), whereas CGGA has 22 features (2 clinical, 20 molecular), given it contains of a Chinese population with race not included in the database.

#	Type	Name	#	Type	Name	#	Type	Name
1	Clinical	Gender	9	Molecular	CIC	17	Molecular	BCOR
2	Clinical	Age	10	Molecular	MUC16	18	Molecular	CSMD3
3	Clinical	Race	11	Molecular	PIK3CA	19	Molecular	SMARCA4
4	Molecular	IDH1	12	Molecular	NF1	20	Molecular	GRIN2A
5	Molecular	TP53	13	Molecular	PIK3R1	21	Molecular	IDH2
6	Molecular	ATRX	14	Molecular	FUBP1	22	Molecular	FAT4
7	Molecular	PTEN	15	Molecular	RB1	23	Molecular	PDGFRA
8	Molecular	EGFR	16	Molecular	NOTCH1	24	Class	Grade

**Table 3:** The effects of using feature selection methods on the TCGA dataset.

ML-ACC	Without FS	LASSO	mRMR
**SVM**	86.769	87.007	74.733
**LR**	86.414	86.414	85.935
**KNN**	82.837	83.313	82.839
**RF**	82.841	82.362	81.886
**AdaBoost**	85.339	85.101	84.621

**Table 4:** The effects of using feature selection methods on the CGGA dataset.

ML-ACC	Without FS	LASSO	mRMR
**SVM**	76.564	76.915	73.085
**LR**	76.570	76.921	76.933
**KNN**	74.816	76.576	71.670
**RF**	74.840	72.741	73.442
**AdaBoost**	74.834	72.033	76.576

**Table 5:** Average performance results (i.e., ACC %, CV = 5) obtained utilizing both LASSO and mRMR-based feature selection with feature-weighting methods on the TCGA dataset.

k	# of Features	SVM	LR	KNN	RF	AdaBoost
15	4	85.340	86.054	84.626	80.100	84.264
14	4	85.340	86.054	84.626	80.100	84.264
13	4	85.340	86.054	84.626	80.100	84.264
12	5	85.102	85.816	84.983	80.814	84.502
11	6	85.698	85.816	84.627	81.172	84.859
**10**	**13**	**87.007**	86.890	84.983	82.481	84.862
9	**13**	**87.007**	86.890	84.983	82.481	84.862
8	18	87.007	86.533	82.599	82.481	85.577
7	18	87.007	86.533	82.599	82.481	85.577
6	20	86.768	86.533	82.479	82.484	85.458
5	20	86.768	86.533	82.479	82.484	85.458
4	22	86.768	86.414	82.718	82.603	85.339
3	22	86.768	86.414	82.718	82.603	85.339
2	23	86.769	86.414	82.837	82.244	85.339
1	23	86.769	86.414	82.837	82.244	85.339

**Table 6:** Average performance results (ACC %, CV = 5) obtained utilizing both LASSO and mRMR-based feature selection with feature-weighting methods on the CGGA dataset.

k	# of Features	SVM	LR	KNN	RF	AdaBoost
15	4	79.371	78.669	74.477	74.476	77.278
14	4	79.371	78.669	74.477	74.476	77.278
13	4	79.371	78.669	74.477	74.476	77.278
**12**	**5**	**80.412**	79.014	75.178	75.886	76.225
11	**5**	**80.412**	79.014	75.178	75.886	76.225
10	8	80.073	79.716	76.219	73.799	76.231
9	8	80.073	79.716	76.219	73.799	76.231
8	10	79.722	76.921	75.517	75.535	74.840
7	10	79.722	76.921	75.517	75.535	74.840
6	11	76.219	77.623	75.535	72.396	74.834
5	11	76.219	77.623	75.535	72.396	74.834
4	13	76.915	76.921	75.173	73.799	73.781
3	14	76.915	77.272	74.822	75.892	73.073
2	16	76.915	77.272	75.523	72.752	72.371
1	16	76.915	77.272	75.523	72.752	72.371

**Table 7:** Comprehensive average performance results (CV = 5) obtained by observing the effects of the feature selection and feature-weighting methods on the TCGA dataset.

	Without FS	With FW and FS		Without FS	With FW and FS	
**ML**	**ACC%**			**AUC**		
**SVM**	86.769	87.007		0.904	0.911	
**LR**	86.414	86.890		0.918	0.923	
**KNN**	82.837	84.983		0.893	0.906	
**RF**	82.841	82.481		0.897	0.900	
**AdaBoost**	85.339	84.862		0.905	0.908	
	**Without FS**	**With FW and FS**		**Without FS**	**With FW and FS**	
**ML**	**F1**			**PRE**		
**SVM**	0.852	0.855		0.801	0.804	
**LR**	0.847	0.852		0.805	0.808	
**KNN**	0.802	0.826		0.782	0.802	
**RF**	0.793	0.792		0.796	0.786	
**AdaBoost**	0.832	0.829		0.803	0.789	
	**Without FS**	**With FW and FS**		**Without FS**	**With FW and FS**	
**ML**	**REC**			**SPEC**		
**SVM**	0.912	0.915		0.837	0.839	
**LR**	0.897	0.905		0.843	0.845	
**KNN**	0.827	0.856		0.832	0.846	
**RF**	0.796	0.802		0.853	0.842	
**AdaBoost**	0.869	0.878		0.845	0.830	

**Table 8:** Comprehensive average performance results (CV = 5) by observing the effects of the feature selection and feature-weighting methods on the CGGA dataset.

	Without FS	With FW and FS		Without FS	With FW and FS
**ML**	**ACC%**			**AUC**	
**SVM**	76.564	80.412		0.815	0.798
**LR**	76.570	79.014		0.792	0.788
**KNN**	74.816	75.178		0.772	0.753
**RF**	74.840	75.886		0.758	0.767
**AdaBoost**	74.834	76.225		0.759	0.749
	**Without FS**	**With FW and FS**		**Without FS**	**With FW and FS**
**ML**	**F1**			**PRE**	
**SVM**	0.609	0.679		0.759	0.807
**LR**	0.633	0.656		0.706	0.788
**KNN**	0.555	0.577		0.743	0.706
**RF**	0.592	0.629		0.659	0.663
**AdaBoost**	0.625	0.603		0.661	0.717
	**Without FS**	**With FW and FS**		**Without FS**	**With FW and FS**
**ML**	**REC**			**SPEC**	
**SVM**	0.527	0.607		0.901	0.913
**LR**	0.584	0.582		0.862	0.907
**KNN**	0.454	0.516		0.908	0.880
**RF**	0.549	0.610		0.855	0.835
**AdaBoost**	0.605	0.536		0.829	0.888

**Table 9:** Comparison with the related methods in the literature for glioma grading tasks on the datasets employed.

Dataset	TCGA		CGGA	
**Total # of Features**	**23**		22	
**Study**	**Our Method**	**[4]**	**Our Method**	**[4]**
**Selected # of Features**	13	14.9	5	17.6
**ACC %**	87.007	87.606	80.412	79.668
**Study**	**Our Method**		**[4]**	
**Method**	mRMR + LASSO		Hierarchical voting-based ensemble scheme	
**Advantages**	Effective, more realistic, and consistent results, and identified feature names		The method employs an ensemble procedure	

**Table 10:** The 13 features were identified using GradWise. Features that emerged in both TCGA and CGGA are shown in bold.

Feature	Frequency of Mutated Genes in TCGA in GBM [37]	Somatic Genomic Alterations in GBM [33]	% GBM Patients Harboring Specific Oncogenic Mutations in TCGA [42]	Mutation Landscape of LGG [32]	Current Role in Oncology		Mechanistic Connections
					**Literature Evidence**	**Use in Clinic**	
**Age**	n/a	n/a	n/a	n/a	Age-associated with unfavorable neuropathological and radiological features in gliomas [43]	Yes, for clinical decision-making via recursive partitioning criteria	Investigational
**IDH1**/IDH2	n/a	n/a	3%	77%	IDH mutation in glioma: molecular mechanisms and therapeutic targets [44,45]	Yes, for tumor molecular characterization	HIF-1α
**PTEN**	34%	31%	19%	n/a	Identification of the Prognostic Signatures of Glioma With Different PTEN Status [34]	Yes, for tumor molecular characterization	TP53, GRIN2A
**NF1**	11%	11%	9%	n/a	An Update on Neurofibromatosis Type 1-Associated Gliomas [36]	Yes, for clinical decision-making and management discussion	EGFR, PTEN
EGFR	26%	26%	15%	6%	Updated Insights on EGFR Signaling Pathways in Glioma [46]	Yes, for tumor molecular characterization	NOTCH1
TP53	34%	29%	16%	46%	Genetic and histologic spatiotemporal evolution of recurrent, multifocal, multicentric and metastatic glioblastoma [35]	Yes, for tumor molecular characterization	PTEN, GRIN2A
PIK3R1	18%	11%	6%	n/a	Somatic Mutations of PIK3R1 Promote Gliomagenesis [47]	Not currently used in the clinic	PI3K
ATRX	n/a	6%	5%	33%	The Role of ATRX in Glioma Biology [48]	Yes, for tumor molecular characterization	ATM
PDGFRA	n/a	4%	5%	n/a	High frequency of PDGFRA and MUC family gene mutations in diffuse hemispheric glioma, H3 G34-mutant: a glimmer of hope? [39]	Investigational	MUC16
NOTCH1	n/a	n/a	n/a	n/a	Oncogenic and Tumor-Suppressive Functions of NOTCH Signaling in Glioma [49]	Investigational	EGFR
GRIN2A	n/a	n/a	4%	n/a	Somatic mutation of GRIN2A in malignant melanoma results in loss of tumor suppressor activity via aberrant NMDAR complex formation [35]	Investigational	PTEN, TP53
MUC16 (CA-125)	11%	n/a	n/a	n/a	MUC16 mutation is associated with tumor grade, clinical features, and prognosis in glioma patients [38]	Used as a serum biomarker in ovarian cancer with implications for other cancers as well [50]	PDGFRA
CIC	n/a	n/a	n/a	20%	CIC protein instability contributes to tumorigenesis in glioblastoma [40]	Not currently used in clinic	EGFR

## Data Availability

The data in this paper has been provided from The Cancer Genome Atlas (TCGA) Research Network (https://www.cancer.gov/tcga, accessed on 5 July 2023) and the Chinese Glioma Genome Atlas (CGGA) (http://www.cgga.org.cn/, accessed on 12 November 2022).

## Supplementary Materials

The following supporting information can be downloaded at: https://www.mdpi.com/article/10.3390/cancers15184628/s1, Figure S1: Expression of mutation features in TCGA dataset; Figure S2: Gene expression profiles in TCGA by primary diagnosis for the 13 features identified (underlined) using GradWise.

## Abbreviations

ACC	Accuracy
AdaBoost	Adaptive Boosting
AUC	Area Under the ROC Curve
CGGA	Chinese Glioma Genome Atlas
CNS	Central Nervous System
F1	F-Measure
GBM	Glioblastoma Multiforme
HGG	High-Grade Glioma
IDH	Isocitrate Dehydrogenase
IPA	Ingenuity Pathway Analysis
KNN	K Nearest Neighbors
LGG	Low-Grade Glioma
LASSO	Least Absolute Shrinkage and Selection Operator
LR	Logistic Regression
mRMR	Minimum Redundancy—Maximum Relevance
NCI	National Cancer Institute
NIDAP	NIH Integrated Data Analysis Platform
NIH	National Institutes of Health
PRE	Precision
REC	Recall
RF	Random Forest
ROC	Receiver Operating Characteristics
RT	Radiation Therapy
SPEC	Specificity
SVM	Support Vector Machine
TCGA	The Cancer Genome Atlas
TMZ	Temozolomide
WHO	World Health Organization
