# Supplementary material for: GradWise: A Novel Application of a Rank-Based Weighted Hybrid Filter and Embedded Feature Selection Method for Glioma Grading with Clinical and Molecular Characteristics
Source: Cancers (Basel). 2023 Sep 19;15(18):4628. doi: 10.3390/cancers15184628 (PMC10526509; doi:10.3390/cancers15184628)
Supplement: Supplementary file 1 [file cancers-15-04628-s001.zip › cancers-2519650-supplementary.pdf]

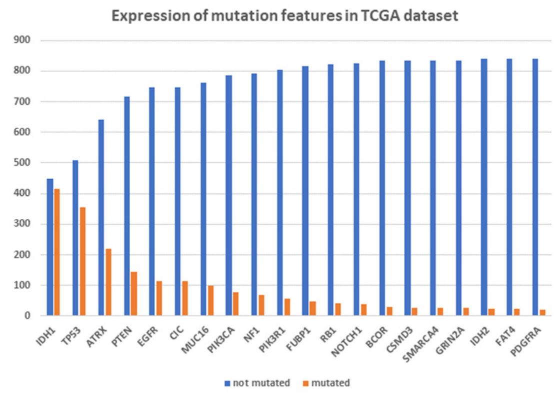

Figure S1. Expression of mutation features in TCGA dataset.

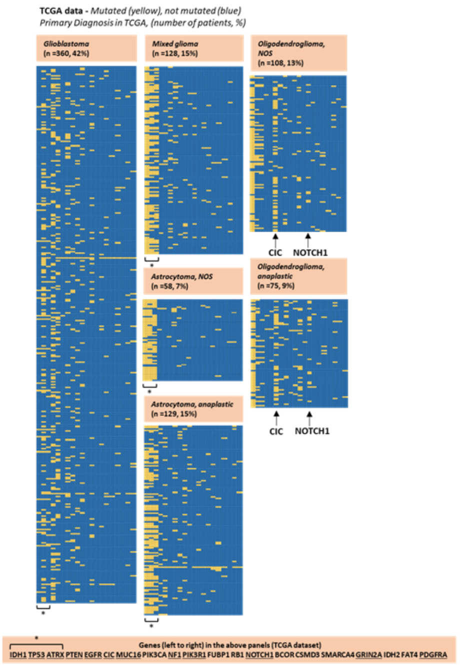

Figure S2. Gene expression profiles in TCGA by primary diagnosis for the 13 features identified (underlined) using GradWise.
